# Impact of Acute and Chronic Amyloid-β Peptide Exposure on Gut Microbial Commensals in the Mouse

**DOI:** 10.3389/fmicb.2020.01008

**Published:** 2020-05-20

**Authors:** Malena dos Santos Guilherme, Hristo Todorov, Carina Osterhof, Anton Möllerke, Kristina Cub, Thomas Hankeln, Susanne Gerber, Kristina Endres

**Affiliations:** ^1^Department of Psychiatry and Psychotherapy, University Medical Center, Johannes Gutenberg University Mainz, Mainz, Germany; ^2^Faculty of Biology, Institute for Developmental Biology and Neurobiology, Center of Computational Sciences Mainz, Johannes Gutenberg University Mainz, Mainz, Germany; ^3^Institute for Human Genetics, University Medical Center, Johannes Gutenberg University Mainz, Mainz, Germany; ^4^Fresenius Kabi Deutschland GmbH, Oberursel, Germany, Oberursel, Germany; ^5^Institute of Organismic and Molecular Evolution, Molecular Genetics and Genome Analysis, Johannes Gutenberg University Mainz, Mainz, Germany

**Keywords:** Alzheimer’s disease, microbiome, anti-microbial, Amyloid-β peptide, mouse model, 5xFAD

## Abstract

Alzheimer’s disease (AD) is the most common form of dementia. Besides its cognitive phenotype, AD leads to crucial changes in gut microbiome composition in model mice and in patients, but the reported data are still highly inconsistent. Therefore, we investigated chronic effects of AD-characteristic neurotoxic amyloid-β (Aβ) peptides as provided by transgenic overexpression (5xFAD mouse model) and acute effects due to oral application of Aβ on gut microbes. Astonishingly, one-time feeding of wild type mice with Aβ_42_ provoked immediate changes in gut microbiome composition (β diversity) as compared to controls. Such obvious changes were not observed when comparing 5xFAD mice with wild type littermates. However, acute as well as chronic exposure to Aβ significantly affected the abundance of numerous individual operational taxonomic units. This provides first evidence that acute *in vivo* exposure to Aβ results in a shift in the enteric microbiome. Furthermore, we suggest that chronic exposure to Aβ might trigger an adaptive response of gut microbiota which could thereby result in dysbiosis in model mice but also in human patients.

## Introduction

Improvements in our healthcare system have led to a global increase in the percentage of elderly people. Currently, nine percent of the population in the world is aged 65 or older; in 2050 this number will increase approximately to 16 percent and the older people will outnumber adolescents aged 15 to 24 years (United Nations Department of Economic and Social Affairs, 2019). Currently, 50 million people suffer from dementia (Alzheimer Disease International, 2019). The most common form of these divergent disorders is Alzheimer’s disease (AD) and age is the major risk factor for developing AD (Alzheimer Disease International, 2019). One of the characteristic hallmarks in the brain of AD patients are amyloid plaques derived from amyloid-β (Aβ) peptides – a cleavage product of the amyloid precursor protein (APP). APP can be processed in two different ways: (1) cleavage by α-secretase gives rise to non-amyloidogenic APPs-α and (2) cleavage by β-and γ-secretase generates neurotoxic Aβ peptides (for detailed reviews see Rossner et al., 2006; Endres and Deller, 2017). Imbalances of this processing or changes in Aβ degradation lead to oligomerization of neurotoxic Aβ peptides which are presumed to be the cause of sporadic AD (Hardy and Higgins, 1992; Yang et al., 2003; Roberts et al., 2017; Lane et al., 2018). Besides this, formation of neurofibrillary tangles of hyperphosphorylated tau protein and a loss of synapses as well as neurons are characteristics of the disease (Whitehouse et al., 1982; Kowall and Kosik, 1987; Shin et al., 1989). In sum, these pathological hallmarks are responsible for the observed decline in cognitive and general abilities in AD patients and finally lead to death. So far, there is no preventive or curative therapy. Treatment with, e.g., Rivastigmine, which acts as an acetylcholinesterase inhibitor, can help to ameliorate disease progression, but cannot stop it (Farlow et al., 2005). The etiology of sporadic AD is not yet fully understood; therefore a more systemic view – including other organs than CNS and also their microbiome – could provide new therapeutic targets.

Only recently, the interplay between brain and gut has gained more attention for researchers regarding neurodegenerative diseases (Quigley, 2017). However, investigations in the field of AD are still rare (for a detailed review see Endres, 2019). Research in the field of, e.g., Parkinson’s disease (PD), a dementia with α-synuclein deposition in analogy to Aβ deposits in AD, is far more advanced and already gave insights into relevant gut-brain-connections. For example, differences in microbial composition of the gut, such as reduced Bacteroidetes and increased Enterobacteriaceae, were found when comparing fecal samples from PD patients with samples of healthy controls (Unger et al., 2016). Simultaneously, about 80% of PD patients suffer from gastrointestinal dysfunction (Poewe, 2008; Cersosimo and Benarroch, 2012). It has also been shown that gut microbiota can directly influence disease progression in PD model mice by regulating motor deficits and neuroinflammation (Sampson et al., 2016). Furthermore, α-synuclein, the main driver of the disease which was also found in gut tissue, is discussed as a biomarker for pre-motor PD by using colon biopsies (Shannon et al., 2012). These investigations give profound basis for the assumption that a neurodegenerative disease might derive from or be influenced by gastrointestinal properties. The avenues which are used for this are not fully understood so far.

Brain and gut are connected *via* the so-called gut-brain-axis which consists of the enteric nervous system (ENS), the *Nervus vagus*, immune cells and the microbiome (Mayer, 2011). The *Nervus vagus* allows bidirectional communication *via* efferent and afferent fibers (Quan and Banks, 2007). The connection between brain and gut enforces speculation if the microbiota and their metabolites may influence AD progression or vice versa if AD pathogenesis impacts the microbiome.

Joachim et al. (1989) already showed Aβ deposition in the gut of individual patients. Also, antimicrobial properties of Aβ on single microbial strains have been demonstrated (Soscia et al., 2010). Furthermore, different studies concluded, that transgenic AD model mice as well as AD patients present differences in microbiota composition in comparison to healthy controls (Brandscheid et al., 2017; Cattaneo et al., 2017; Harach et al., 2017; Shen et al., 2017; Vogt et al., 2017; Bauerl et al., 2018). The investigations of the role of microbiota in AD are still highly inconsistent. Harach et al. (2017), for example, showed an increase of Firmicutes, but a decrease of Bacteroidetes, in fecal samples of 1 month old AD model mice (APPPS1) as compared to wild type controls. The opposite result could be found in the same mouse strain at an age of 8 months. In a study using fecal samples of 9 week old 5xFAD mice, elevated amount of Firmicutes and less Bacteroidetes were detected as compared to wild type controls (Brandscheid et al., 2017). The investigated mouse strains represent genetic models, which can be used for observatory studies of chronic exposition of gut tissue with neurotoxic Aβ peptides especially because the peptide itself has also been detected in the gut (Brandscheid et al., 2017). However, acute effects have not been investigated so far *in vivo* and compared to the prolonged exposure.

The aim of our study was to investigate chronic as well as acute effects of Aβ exposition in the gut. 5xFAD mice were used as a model for AD, which express mutated human APP and PSEN1 transgenes with a total of five mutations and show Aβ deposition in the brain and gut tissue already at an age of 4 weeks (Oakley et al., 2006; Brandscheid et al., 2017). We analyzed gut bacteria of C57Bl/6J wild type mice in comparison to 5xFAD transgenic littermates by 16S-rDNA sequencing. Additionally, acute effects of Aβ were investigated *in vivo* after oral administration of Aβ peptides to wild type mice. As expected, differences in the gut microbiome could be observed by comparing 5xFAD mice with wild type controls but interestingly, also acute exposition of gut tissue for just 5 h with Aβ peptides (one complete gut passage) led to substantial differences in microbiota as compared to animals treated with a scrambled, non-active peptide.

## Materials and Methods

### Peptides

For the incubation of feces bacteria and oral application to the mice, human Aβ_1__–__42_ or scrambled, biologically inactive peptide (both AnaSpec, Seraing, Belgium) in 5 mM NH_4_OH buffered in PBS was used. TAMRA-labeled peptide (AnaSpec, Seraing, Belgium) was used for detecting gut transition of Aβ.

### Quantitation of Colony Forming Units and Bacterial Viability Assay

#### Anaerobic Cultivatable Community

Freshly collected fecal pellets were suspended in isotonic sodium chloride solution (0.9%, 100 μl/mg) using a hand-held electric stirrer (Xenox, Fähren, Germany). Aliquots of 50 μl were supplemented with 5 μl of peptide solution in 0.4 mM NH_4_OH buffered in PBS. After 10 min incubation at room temperature under careful mixing for every 2 min, 20 μl of this suspension were added to 4 ml of thioglycollate bouillon (Becton Dickinson GmbH, Heidelberg, Germany) and kept for overnight at 4°C. The following day, 1 μl of the diluted fecal suspension was spread on Schaedler agar plates (Becton Dickinson GmbH, Heidelberg, Germany) and incubated for 48 h anaerobically (Anoxomat, Mart Microbiology B.V, Drachten, Netherlands). Lastly, colony forming units (CFU) were counted and normalized to controls from the same donor mouse incubated with scrambled peptide.

#### Family-Specific Cultivation

For assessing toxic effects of Aβ on specific microbial families, freshly collected feces was suspended and incubated with 2 μM peptide solution as described above. Afterward, feces suspensions were diluted with 0.9% sodium chloride solution for plating 1 ml on 3M^TM^-Petrifilm plates specific for Enterobacteriaceae and Lactobacillaceae (3M Deutschland GmbH, Neuss, Germany). Plates were incubated for 20 h at 37°C. CFU were counted and normalized to samples from the same donor mouse incubated with scrambled peptide.

#### Viability Assay

After incubation of diluted feces samples with Aβ peptide or scrambled control for plating (see above), additional 1:100 dilutions were prepared and viability measured by using the BacTiter Glo assay as indicated by the vendor (Promega, Mannheim, Germany). Relative luminescence was measured and normalized to controls from the same donor mouse incubated with scrambled peptide.

### Animals

B6SJL-Tg(APPSwFlLon,PSEN1^∗^M146L^∗^L286V)6799Vas/Mmjax (5xFAD) mice (Jackson Lab, Bar Harbor, Maine, United States) were maintained by crossbreeding with C57BL/6J background as described in Reinhardt et al. (2018). Animals aged 9–10 weeks were used and non-transgenic offspring served as control and for acute treatment with peptides. All animals were group-housed with 3–5 animals per cage in a 12 h light/dark cycle with food (Ssniff Spezialdiäten GmbH, Soest, Germany) and water available *ad libitum*. At least one week before sampling of feces or chyme, animals were single-caged to avoid microbial transfer, e.g., by coprophagy. All procedures were performed in accordance with the European Communities Council Directive regarding care and use of animals for experimental procedures and were approved by local authorities (Landesuntersuchungsamt Rheinland-Pfalz; approval number G17-1-035).

### Oral Peptide Application

Animals were lightly anesthetized with Isoflurane and 10 μg of peptide or solvent (10 mM NH_4_OH) administered slowly into the oral cavity. Mice were kept in manual fixation up to ingestion of the fluid to prevent aspiration.

### Collection of Chyme

Animals were deeply anesthetized with isoflurane and sacrificed by decapitation. For microbiome determination, feces pellets close to the rectum were taken. For assessing fluorescence after feeding of TAMRA-labeled Aβ, the whole gut was placed in ice cold isotonic sodium chloride to prevent further peristalsis movement. Segments of 2 cm length were then chopped starting from the stomach, the content collected by longitudinal opening and stored on ice until further use.

### Western Blot and Fluorescence Detection

To measure the stability of TAMRA-labeled Aβ in the gastrointestinal milieu, chyme samples were collected as described before and diluted with 500 μl ice cold PBS. TAMRA-labeled Aβ (0.025 μM or 0.005 μM) was added to chyme or PBS as control and incubated for 2 h at 37°C with gently mixing every 20 min. Afterward, LDS NuPAGE buffer (1×, Life Technologies, Carlsbad, California, United States) and DTT (1 M, 10% v/v, Carl Roth GmbH & Co. KG, Karlsruhe, Germany) were added and the solution was heated for 10 min at 70°C. Proteins were separated on a 12%-SDS polyacrylamide gel and subsequently transferred to a nitrocellulose membrane (GE Healthcare, Chalfont, Great Britain). The membrane was blocked with I-block solution (0.2% in PBS/T, Thermo Fisher Scientific) for 1 h before incubation with primary antibody in a dilution of 1:1,000 overnight at 4°C. On the next day, the membrane was washed with PBS/T and incubated with the secondary antibody coupled with horse reddish peroxidase (Thermo Scientific, Chalfont, Great Britain). Chemiluminescent signals were detected after application of SuperSignal West Femto chemiluminescent substrate (Thermo Scientific, Chalfont, Great Britain) by using a CCD-camera (Stella 3200 Camera, Raytest, Straubenhardt, Germany). Fluorescence signal was detected by using the Storm Scanner 860 (Molecular Dynamics, Caesarea, Israel).

### Assessment of Gut Transition Time of Peptides

After oral application of TAMRA-labeled Aβ or NH_4_OH as control and sacrifice of the animals 2, 4, or 24 h later, chyme samples were diluted with 500 μl ice cold PBS, incubated for 5 min and centrifuged. Fluorescence was measured by using 50 μl of the supernatant in duplicates (Exc 540 nm and Em 580 nm, FLUOstar Optima, BMG Labtech GmbH, Ortenberg, Germany) at intervals of 1 min over a period of 1 h at 37°C with shaking before every measurement. The mean of the values of the measurements between 15 and 45 min was used to calculate the difference between the TAMRA-labeled Aβ-treated animals and solvent-treated control animals.

### DNA Extraction From Fecal Samples

Fecal samples were stored at −80°C before DNA extraction was done by using the QIAmp Fast DNA Stool Mini Kit (Qiagen GmbH, Hilden, Germany) as recommended by the vendor.

### Sequencing and Taxonomic Assignment of 16S rDNA Amplicons

Library preparation and Illumina sequencing was performed by StarSEQ GmbH (Mainz, Germany). For amplicon generation, the V3-V4 16S metagenomics sequencing system (Illumina) was used. Amplicons were paired-end sequenced (2 × 300 bp) on an Illumina MiSeq platform. De-multiplexed sequences were loaded into QIIME2 version 2018.6 (Caporaso et al., 2010) and de-noising was done using the DADA2 algorithm (Callahan et al., 2016). Due to a low quality of the reverse reads, neither read merging within QIIME nor external read merging with BBMerge from the BBMap suite^1^ retained a sufficient amount of reads, so we decided to exclude the reverse reads from downstream analysis. Forward reads were truncated to 255 bp after quality control. A naïve Bayes classifier was trained on Greengenes database 13_8 containing the V3–V4 region only with QIIME’s internal implementation of scikit-learn. Taxonomic assignment was then done on 97% sequence identity. A multiple sequence alignment was done with MAFFT and a phylogenetic tree was calculated using the q2-phylogeny plugin.

### Microbial Composition of Fecal Samples

We employed the *phyloseq* R package for downstream analysis of 16S data. Separate statistical comparisons were always performed between wild type vs 5xFAD and scrambled Aβ vs Aβ-fed animals, respectively. Alpha diversity was estimated using the Chao and Shannon index. In order to account for different library sizes, each sample was normalized to the smallest library size by random sampling with replacement. This process was repeated a 1,000 times and the average values from all runs were considered as final estimates for alpha diversity. Groups were compared statistically with an unpaired *t*-test. Differences in beta diversity were analyzed using canonical analysis of principal coordinates (CAP) (Anderson and Willis, 2003) as implemented in the *vegan* R package. Ordinations based on both the Bray–Curtis dissimilarity as well as the binary Jaccard distance were performed to investigate quantitative and qualitative differences in beta diversity. Community composition at the phylum and family level was compared based on relative abundance. Subsequently, we performed differential abundance analysis by estimating log2 fold changes (FC) of bacterial abundance with the DESeq2 package (Love et al., 2014). This method was chosen because DESeq2 was previously shown to offer increased sensitivity to detect differentially abundant operational taxonomic units (OTUs) while keeping false discovery rates below 0.05 with small sample sizes (Weiss et al., 2017). Briefly, absolute bacterial abundances were transformed to normalized counts using the median-of-ratios method implemented in DESeq2. FC of bacterial abundance were calculated using generalized linear models assuming a negative binomial distribution. The relationship between the mean and dispersion parameters was estimated using local regression. The significance of FC was evaluated with Wald *z* tests. Differences were considered statistically significant if the adjusted *p*-value of the respective FC was below 0.05. *p*-values are two-tailed and adjustment for multiple comparisons was performed by controlling the false discovery rate with the help of the Benjamini–Hochberg method.

### Statistical Analysis

**FIGURE 1:**
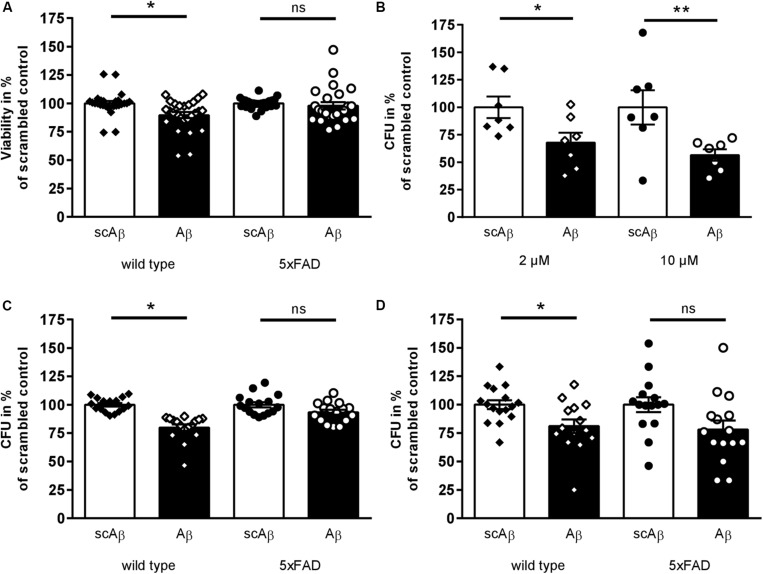
Toxic effects of synthetic Aβ_42_ on murine fecal microbial organisms. **(A)** Fecal samples of transgenic 5xFAD mice and wild type littermates were diluted and incubated with Aβ_42_ peptide or scrambled control (2 μM) for 10 min. Viability was measured and calculated in percent of scrambled peptide treated samples (*n* = 13 animals per group,♂ = 6–7, ♀ = 6–7). **(B)** Fecal material suspension from wild type mice were incubated with 2 or 10 μM Aβ_42_ or the respective amount of scrambled peptide. Subsequently, aliquots of further diluted bacteria suspension were plated on Schaedler agar and incubated anaerobically for 48 h at 37°C. Number of colony forming units (CFU) of samples treated with scrambled peptide control were set to 100% (*n* = 7 animals per group; ♂ = 3, ♀ = 4). **(C, D)** After incubation of fecal samples from transgenic or wild type mice with 2 μM Aβ_42_ or scrambled peptide, suspensions were spread on plates selective for Lactobacillaceae **(C)** and Enterobacteriaceae **(D)** (*n* = 10–11 per group, ♂ = 5–6, ♀ = 5). Datapoints for individual normalized measurements are shown in each graph (*p < 0.05; **p < 0.01).

All statistical analyses were performed using GraphPad Prism 6 for Windows or R version 3.5. Data are graphically represented as mean + standard error of the mean (SEM) (Figure 1) or with boxplots and scatter plots showing individual values (Figure 3). Statistical analysis was performed with an unpaired two-tailed Student’s *t*-test (^∗^*p* < 0.05, ^∗∗^*p* < 0.01, and ^∗∗∗^*p* < 0.001) if not stated otherwise.

## Results

### Impact of Aβ on Viability of Murine Fecal Bacteria

An antimicrobial effect of Aβ_42_ on single microbial species such as *Enterococcus faecalis* or *Candida albicans* has been shown before (Soscia et al., 2010). In order to ensure the toxic properties of Aβ peptides in complex microbiota mixtures, fecal samples from 5xFAD mice and wild type littermates were incubated with Aβ_42_ or scrambled peptide as control (incubation with solvent only did not statistically deviate from scrambled control, data not shown). Ten minutes of incubation sufficed to significantly reduce viability of fecal microorganisms from wild type mice in comparison to scrambled peptide treated samples (Figure 1A). Interestingly, no such toxic effect of the active peptide could be shown within samples derived from transgenic AD model mice. Furthermore, samples derived from wild type mice treated with 2 or 10 μM Aβ_42_ were plated on Schaedler agar plates to analyze the effect on all anaerobically cultivatable bacteria. Two different concentrations were selected to address a wider range of bacteria. CFU were counted after 48 h of incubation. At both concentrations, a significant reduction of about 30–40% of bacterial growth was observed (Figure 1B). To assess if differences in vulnerability due to Aβ exist in different bacterial families, diluted bacterial suspension from transgenic and wild type animals were subsequently plated on agar plates selective for Lactobacillaceae (Figure 1C) or Enterobacteriaceae (Figure 1D). Both families were significantly reduced after Aβ_42_ treatment in comparison to scrambled peptide control when fecal material was derived from wild type mice. However, no significant effects could be observed for Lactobacillaceae from transgenic 5xFAD animals, and only a non-significant reduction of Enterobacteriaceae occurred (*p* = 0.09).

### Gastro-Intestinal Passage of Orally Administered Aβ Peptides

**FIGURE 2:**
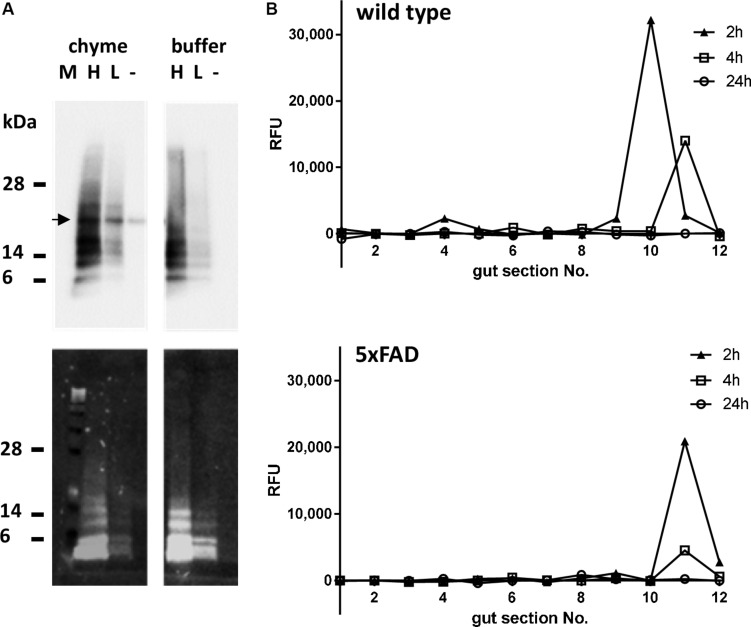
Stability and time-dependent localization of *per os* administered Aβ peptides in the murine gastrointestinal tract. **(A)** TAMRA-labeled Aβ peptide was incubated in two different concentrations [high (H): 0.025 μM; low (L): 0.005 μM] with PBS-diluted gastric content of wild type mice for 2 h at 37°C. Solvent of the peptide was used as a negative control (–). Additionally, samples with PBS instead of chyme were incubated as described (buffer). Proteins were separated on PAA gel and transferred to nitrocellulose. Detection of Aβ-dependent signal was conducted by antibody 6E10 (upper panel) or by fluorescence (lower panel). The arrow indicates a cross reactivity only occurring in chyme. **(B)** To assess the passage of orally administered Aβ peptides, wild type or 5xFAD mice were fed with 10 μg of TAMRA-labeled Aβ peptides and sacrificed at indicated time points. TAMRA-dependent fluorescence was determined in the chyme of 2 cm gut sections numbered from 1 (oral end = stomach) to 12 (rectal section). Values are presented as measured difference between the TAMRA-Aβ-treated animals and solvent-treated control animals (one animal per time point, technical duplicates).

Before oral treatment of the mice, the stability of the Aβ peptide in the gastro-intestinal milieu had to be proven. Therefore, TAMRA-labeled Aβ_42_ was incubated with PBS-diluted content of the stomach of wild type mice or PBS as control for 2 h at 37°C (exponential decay constant of stomach emptying in mice: 74 min; Schwarz et al., 2002). Detection of Aβ-dependent signals by Western blotting using the antibody 6E10 showed similar patterns of Aβ monomer and oligomers incubated with gastric content or PBS, independent of Aβ concentration (Figure 2A, upper panel). Fluorescence detection supported a clear assignment of the TAMRA-labeled peptide as the same pattern could be obtained as with the antibody (Figure 2A, lower panel). In sum, this indicated that the peptide would at least be stable within a 2 h period of digestive passage. After the stability of the Aβ peptide in the gastrointestinal milieu could be ensured, mice were orally treated with the TAMRA-labeled peptide and sacrificed after 2, 4, and 24 h. To measure the transit time of the peptide, the gut was divided into 2 cm segments and fluorescence was measured within diluted chyme. Two hours after treatment, the TAMRA-labeled peptide could be detected in the 10^th^ segment of the gut of the wild type animal (according to cecum) and in the 11^th^ segment of the transgenic one (Figure 2B). Two hours later, in both animals the signal was measured in the 11^th^ segment (cecum ampulla). No signal could be detected in any sample 24 h after the treatment. For further experiments, a complete gut passage was suspected after 5 h in mice of respective age and strain which fits to earlier reports on C57Bl/6 mice (Schwarz et al., 2002).

**FIGURE 3:**
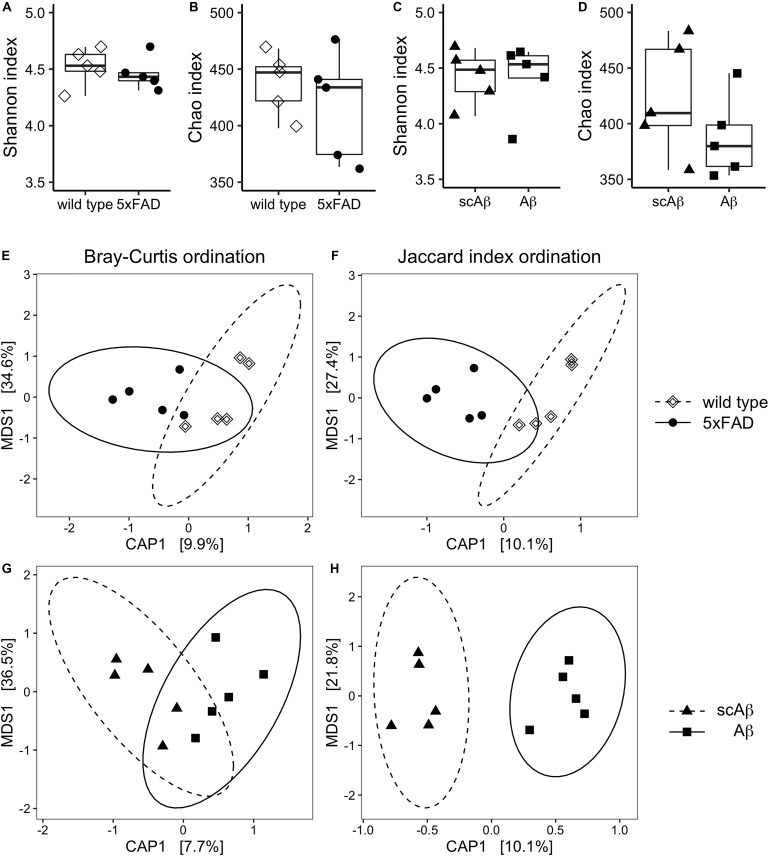
Diversity measures of gut microbiota following chronic or acute exposure to Aβ. Species richness and evenness of each experimental group were evaluated by estimating the Chao index and Shannon index, respectively. Panels **(A)** and **(B)** show results for wild type compared to 5xFAD mice. Differences in alpha diversity between wild type animals receiving scrambled Aβ (scAβ) or Aβ are depicted in panels **(C)** and **(D)**. Data are shown as boxplots and individual values. Beta diversity was evaluated by calculating Bray–Curtis dissimilarity **(E, G)** and binary Jaccard distance **(F, H)**. Results were visualized using canonical analysis of principal coordinates (CAP) for each dissimilarity measure separately. Since there are two groups in each ordination analysis, only one constrained dimension is calculated (CAP1), which is shown on the *x*-axis. The *y*-axis corresponds to the first unconstrained dimension (MDS1). Confidence ellipses around the respective group centroid were drawn at the 95% confidence level. The percentage of total inertia captured by each axis is shown in brackets. MDS: Multi-dimensional scaling.

### Diversity of the Gut Microbiome Under Chronic and Acute Exposure to Aβ Peptides

We employed high-throughput sequencing of the bacterial 16S rRNA gene in order to investigate the impact of acute or chronic exposure to the toxic Aβ peptide on the murine gut microbiome *in vivo*. Therefore, feces from 5xFAD mice and wild type littermates as well as feces from wild type mice treated with Aβ or scrambled Aβ were collected. Subsequently, DNA was purified and subjected to 16S rDNA sequencing. All animals used in these experiments were female.

In the first step of the analysis we compared the alpha and beta diversity of the negative control sample to all experimental groups as means of quality control. Estimates of species richness as well as evenness were considerably lower for the negative control sample as indicated by the Chao and Shannon index, respectively (Supplementary Table S1). Furthermore, unconstrained analysis of principal coordinates based both, the Bray-Curtis dissimilarity and Jaccard index, revealed that the negative control was distinctly separated from all experimental groups in reduced space (Supplementary Figure S1). This accounted for a large amount of the dispersion in sample scores, therefore the negative control was removed from the subsequent downstream analysis in order to better represent differences between experimental groups.

We did not observe differences in the alpha diversity estimates between wild type and 5xFAD animals in terms of species evenness (Figure 3A) and richness (Figure 3B). The comparison of mice fed with scrambled Aβ or the active form of the peptide yielded similar results with a very slight non-significant trend of reduced species richness in the Aβ group (mean Chao index = 423.4 vs 387.9 in scrambled Aβ compared to Aβ-fed animals, *p* = 0.24, Figures 3C,D).

Comparison of beta diversity of 5xFAD vs wild type mice using CAP resulted in better separation of experimental groups along the constrained dimension based on the Jaccard index (Figures 3E,F). Nevertheless, experimental group assignment only explained a small amount of the dispersion between samples and the grouping effect was not significant. In contrast, beta diversity significantly differed between Aβ- and scrambled Aβ-fed animals in the Jaccard distance based ordination. This was indicated by non-overlapping 95% confidence ellipses (Figure 3H). The difference was not significant when beta diversity was measured with the Bray–Curtis dissimilarity, implying that the shift in microbiota composition was qualitative in nature (Figure 3G).

### Community Composition of Gut Microbiome

**FIGURE 4:**
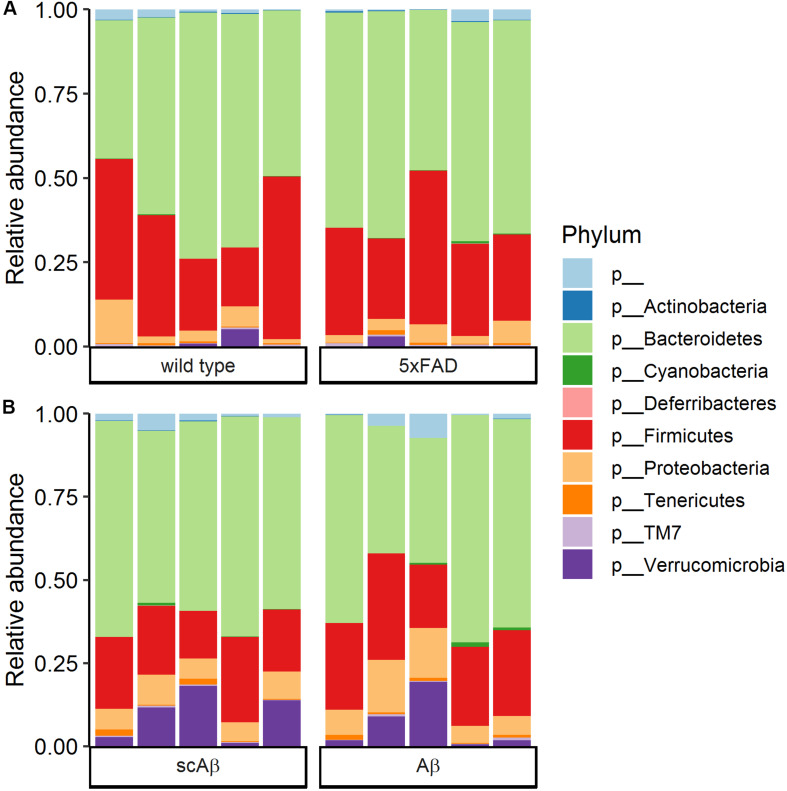
Community composition at the phylum level. Bars show the relative abundance of bacterial phyla in **(A)** wild type compared to 5xFAD mice and **(B)** wild type animals fed with scrambled Aβ (scAβ) versus active Aβ. Phyla with a relative abundance below 0.05% were filtered out from the data set. The label “p__” corresponds to operational taxonomic units that could not be classified to a reference phylum.

Community composition of the gut microbiome at the phylum level was highly similar in 5xFAD mice as compared to wild type littermates (Figure 4A). The most dominant phylum was Bacteroidetes with an average relative abundance around 60%, followed by Firmicutes with an average relative abundance of approximately 30% in both groups. Proteobacteria and Verrucomicrobia were the only remaining classified phyla with a relative abundance of above 1%. The phylum composition of Aβ- compared to scrambled Aβ-fed animals revealed similar trends (Figure 4B). The average relative abundance of Bacteroidetes was slightly lower in the Aβ group (53.9% compared to 59.5% in the scrambled Aβ group, *p* = 0.463). In contrast, Firmicutes average relative abundance of 25.4% in Aβ-fed animals was marginally higher than the scrambled peptide group (20.2%, *p* = 0.102). Nevertheless, differences in relative abundance at the phylum level were not statistically significant. The community composition at the family level is shown in Supplementary Figure S2 for wild type and 5xFAD animals and Supplementary Figure S3 for animals fed with Aβ or the scrambled peptide.

### Differential Abundance Analysis

**FIGURE 5:**
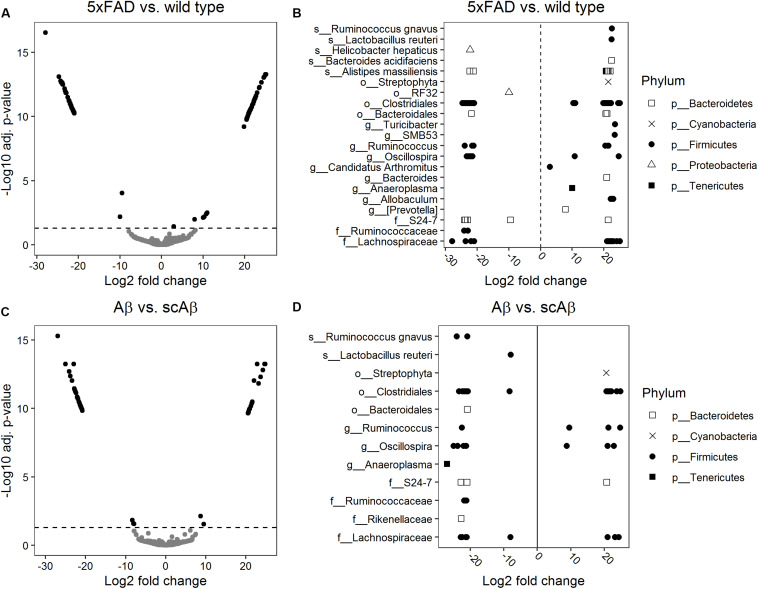
Differential abundance analysis of bacterial operational taxonomic units (OTUs). Log2 fold changes of bacterial abundance were calculated using DESeq2. Volcano plots show the log2 fold changes versus –log10 adjusted *p*-values for 5xFAD relative to wild type mice **(A)** and animals fed with Aβ relative to mice receiving the scrambled peptide **(C)**. OTUs were considered to be differentially abundant if the adjusted *p*-value (Benjamini–Hochberg method) was below 0.05 (indicated by a dashed horizontal line). Significant log2 fold changes appear as black dots in panels **(A)** and **(C)**. The lowest level of taxonomy which could be assigned to the differentially abundant OTUs is depicted in panel **(B)** for 5xFAD mice relative to wild type littermates and in panel **(D)** for mice fed with Aβ relative to the scrambled peptide control group. Log2 fold changes greater than 0 correspond to OTUs significantly enriched in 5xFAD or Aβ-fed mice relative to the respective control group.

We performed a differential abundance analysis on the OTU level by calculating log2 FC of bacterial abundance using the DESeq2 package. Diagnostic plots from this analysis are shown in Supplementary Figure S4. This analysis revealed 109 phylotypes with a significantly different abundance in 5xFAD animals relative to the wild type group (Figures 5A,B). The majority of these OTUs were associated with the Clostridiales order within the Firmicutes phylum. A large amount of the differentially abundant OTUs could not be classified to a reference taxonomy beyond the order and family level. These taxonomic units included OTUs with either significantly increased or decreased abundance in 5xFAD animals which implies different influence of chronic Aβ exposure on the growth of bacteria at lower taxonomic levels such as genera, species, or strains. At the genus level, we detected individual OTUs associated with *Allobaculum*, *Prevotella*, *Anaeroplasma*, *Bacteroides*, *SMB53*, and *Turicibacter*, respectively, all of which demonstrated significantly increased abundance in 5xFAD animals as compared to wild type littermates. At the species level, we observed an enhanced growth of individual OTUs belonging to *Bacteroides acidifaciens*, *Lactobacillus reuteri*, and *Ruminococcus gnavus* in the 5xFAD group. In contrast, abundance of *Helicobacter hepaticus* was significantly decreased relative to wild type animals whereas individual OTUs associated with *Alistipes massiliensis* demonstrated both, increased and decreased abundance in 5xFAD animals (for single fold changes and adjusted *p*-values see Supplementary Table S2).

Our comparison of the abundance of individual OTUs between Aβ- and scrambled Aβ-fed animals resulted in 55 differentially abundant OTUs (Figures 5C,D) most of which belonged to the Clostridiales order. Similarly to the previous analysis, multiple OTUs could not be classified beyond the order and family level. At the genus level, one OTU assigned to *Anaeroplasma* showed significantly decreased abundance in the Aβ group. In contrast, OTUs classified to *Oscillospira* and *Ruminococcus* were associated with instances of both, increased and decreased abundance, relative to the samples derived from scrambled Aβ-fed animals. At the species level, we identified one OTU associated with *Lactobacillus reuteri* and two OTUs belonging to *Ruminococcus gnavus* which demonstrated significantly reduced growth following feeding with Aβ (fold changes and adjusted *p*-values are given in Supplementary Table S3).

## Discussion

The link between AD and alterations in the gut microbiome composition has already been established in several experimental animal models (Brandscheid et al., 2017; Harach et al., 2017; Shen et al., 2017; Zhang et al., 2017; Bauerl et al., 2018; Peng et al., 2018; Zhan et al., 2018) and clinical studies (Cattaneo et al., 2017; Vogt et al., 2017; Zhuang et al., 2018). However, the exact causal mechanism is still not understood. On one hand, perturbations in gut bacterial homeostasis might lead to increased intestinal permeability, low grade inflammation or insulin resistance and obesity (Turnbaugh et al., 2006; Cani et al., 2008, 2009), the last two of which are risk factors for developing AD (e.g., Kivipelto et al., 2005; Fitzpatrick et al., 2009; Luchsinger et al., 2012; Ferreira et al., 2018). On the other hand, it is plausible that shifts in gut bacteria in AD patients might occur as a result of life style and dietary changes after onset of the disease. For instance, in a cross-sectional study, AD patients had an exacerbated nutritional status compared to age-matched controls (Saragat et al., 2012). An alternative mechanism of how the host affects microbiota in the context of AD has been hypothesized by us in a recent *in silico* study (Hewel et al., 2019). In this previous work, we demonstrated that host miRNAs differentially expressed in patients suffering from AD have the potential to bind on key regulatory sequences in commensal microbiota, thereby possibly regulating transcription in bacterial pathways.

Despite representing a major hallmark of AD pathology, the exact physiological function of the Aβ peptide is not known. However, both *in vitro* (Soscia et al., 2010; Kumar et al., 2016; Spitzer et al., 2016) and *in vivo* (Kumar et al., 2016) studies have demonstrated that Aβ exerts anti-microbial properties. Furthermore, oligomerization and fibrillization of the peptide, which are involved in the process of plaque deposition, are also characteristics shared with known anti-microbial peptides (Soscia et al., 2010; Kagan et al., 2012). This has led to the proposal of a new paradigm for the role of Aβ in AD pathogenesis referred to as the antimicrobial protection hypothesis of AD (reviewed in Moir et al., 2018). Considering the fact that Aβ depositions are also found in the intestine, we investigated if acute or chronic exposure to the toxic peptide modulates mouse gut microbiota composition *in vitro* and *in vivo*. Previous *in vitro* experiments on the anti-microbial properties of Aβ were performed under aerobic conditions (Soscia et al., 2010; Spitzer et al., 2016), which is not representative for the predominantly anaerobic environment of the intestine. Therefore, we incubated fecal samples from wild type and 5xFAD mice anaerobically in the current study. Strikingly, Aβ inhibited bacterial growth in fecal samples originating from wild type animals. Only a tendency of bacterial growth reduction of Enterobacteriaceae of 5xFAD mice could be observed. This might suggest that prolonged exposure to the peptide in 5xFAD animals triggers an adaptive response in some gut microbiota, but individual species could still be affected. This assumption is corroborated by *in vitro* experiments showing that Aβ inhibited *E. faecalis* growth at early time points, whereas bacterial growth resumed at later time points (Soscia et al., 2010).

After observing the growth-inhibiting effects of Aβ on fecal bacteria *in vitro*, we also investigated how this translates to the *in vivo* situation. We did not detect any difference in alpha diversity measures between wild type and 5xFAD animals or between wild type mice fed with Aβ or scrambled peptide which is in line with previous reports in mice of similar age (Harach et al., 2017; Shen et al., 2017; Zhang et al., 2017). In contrast, conflicting results have been described regarding longitudinal changes of alpha diversity in mouse models of AD. For instance, Zhang et al. (2017) and Bauerl et al. (2018) observed a significant increase in alpha diversity in APP/PS1 transgenic mice over time whereas Shen et al. (2017) reported a significant decrease in older APP/PS1 mice. Differences were not gender-driven since the two studies showing an increase used male and female mice, respectively. Furthermore, APP/PS1 mice had significantly higher alpha-diversity compared to control animals at 8 months of age in a study by Harach et al. (2017). In the senescence accelerated mouse prone 8 (SAMP8) model of AD, alpha diversity was significantly lower in 7-month old SAMP8 mice as compared to age-matched controls (Zhan et al., 2018). While differences in the mouse models of AD and experimental designs might be partly responsible for these conflicting results, the true association between alpha diversity of the gut microbiome and AD still needs to be elucidated.

5xFAD animals did not significantly differ from wild type littermates in terms of beta diversity which is in agreement with existing studies in mice of comparable age (Harach et al., 2017; Zhang et al., 2017; Bauerl et al., 2018). Remarkably, wild type mice fed with Aβ were clearly separated from animals receiving the scrambled peptide on the ordination plot when beta diversity was measured with the Jaccard distance. This might again be an indication that intestinal bacteria in wild type mice are more susceptible to the acute effects of Aβ, whereas prolonged exposure in 5xFAD mice already led to compositional adaptation. However, longitudinal investigations or studies in older mice consistently reported a difference in beta diversity between mouse models of AD and controls at later times points such as 6, 7, 8, and 24 months of age (Harach et al., 2017; Bauerl et al., 2018; Peng et al., 2018; Zhan et al., 2018). Therefore, we hypothesize that increased Aβ burden might be an early driver of changes in the gut microbiome followed by an adaptive response to the direct anti-microbial effects of the peptide. This is also reflected by the lack of *in vitro* toxic effects of Aβ peptides administered to fecal samples derived from 5xFAD mice. However, the initial perturbations of gut homeostasis might trigger a larger cascade of subsequent events contributing to AD development and long-term alterations in microbiome composition. For instance, we previously described a significantly reduced body weight in male 5xFAD mice compared to wild type controls as early as 6 weeks of age (Brandscheid et al., 2017). Altered gut microbiome is well known in the context of obesity (Turnbaugh et al., 2006; Cani et al., 2008), therefore it is plausible to assume that body weight reduction might also be associated with shifts in fecal bacteria.

With regard to community composition of intestinal bacteria in female mice aged 10 weeks, we did not detect any significant changes at the phylum level. In an earlier study, we described a significantly increased relative abundance of Firmicutes accompanied by a significant decrease in Bacteroidetes in male 5xFAD mice compared to wild type littermates at the age of 9 weeks (Brandscheid et al., 2017). It is important to mention that we previously employed a targeted approach to only quantify a specific subset of phyla and species using qPCR. Here, we performed a more comprehensive microbiome characterization by high-throughput amplicon sequencing of the 16S rRNA gene. Therefore, methodological differences might in part account for the discrepancy in results. However, gender itself is also associated with differences in intestinal microbiota composition (Yurkovetskiy et al., 2013; Fransen et al., 2017; Elderman et al., 2018). Importantly, female mice were reported to have a higher diversity and richness which might be an indicator of a more robust microbiome (Yurkovetskiy et al., 2013; Elderman et al., 2018). Furthermore, male mice exhibited an altered gut bacterial composition at puberty and this was not observed in female animals (Yurkovetskiy et al., 2013). Therefore, shifts at the phylum level in the context of AD might take longer to develop in female mice as compared to males which might explain why we did not detect any changes in the current study. In line with this, Bauerl et al. (2018) described a significantly increased abundance of the phylum Proteobacteria in 6-month-old APP/PS1 female mice compared to controls. The difference was not present at 3 months of age.

Even though the composition of gut microbiota was not altered after acute or chronic exposure to Aβ at higher taxonomic levels, we detected numerous differentially abundant OTUs pointing to early perturbations in gut homeostasis at the species and strain level. In line with the *in vitro* growth inhibiting effect of Aβ on Lactobacillaceae family, we observed a significantly lower abundance of an OTU associated with *Lactobacillus reuteri* in animals fed with Aβ compared to scrambled peptide. Interestingly, abundance of *L. reuteri* was drastically decreased in the offspring of mice kept on a high fat diet compared to mothers on a regular diet in an autism mouse model (Buffington et al., 2016). Moreover, supplementation with this bacterium significantly improved deficits in social behavior in the offspring of the high-fat diet mice. However, we observed an increased abundance of this bacterium in 5xFAD mice compared to wild type littermates, which in theory may again point to a compensatory reaction after a prolonged exposure to the toxic Aβ peptide. However, the possible role of *L. reuteri* in AD needs to be further validated. Another potential target of interest could be *Ruminococcus gnavus* which belongs to the Lachnospiraceae family and has been linked to inflammatory bowel disease (Joossens et al., 2011; Hall et al., 2017; Henke et al., 2019). We identified two OTUs corresponding to this species with significantly lower abundance in mice fed with Aβ, however, one OTU demonstrated an increased abundance in 5xFAD mice relative to wild type controls. Interestingly, OTUs associated with *R. gnavus* were identified to have an increased abundance in patients with clinically diagnosed AD dementia compared to age-matched individuals with normal cognitive function (Zhuang et al., 2018). In the same study, an OTU associated with *Prevotella* was more abundant in the AD group which is in line with our observation of enhanced growth of a *Prevotella* OTU in 5xFAD mice compared to controls. In contrast, a decreased abundance of *Prevotella* was described in AD mice as compared to controls (Shen et al., 2017; Peng et al., 2018), so results remain controversial. An OTU belonging to the *Bacteroides* genus demonstrated an enhanced growth in 5xFAD mice in our study. Increased abundance of this bacterial genus was also described in patients with AD by Vogt et al. (2017). Furthermore, *Bacteroides* abundance in the AD group was positively correlated with Aβ_42_/Aβ_40_ and p-tau/Aβ_42_ in cerebrospinal fluid, both of which are biomarkers for exacerbated AD pathology.

Overall, the abundance of multiple other OTUs, which belong to the same higher taxonomic unit, was regulated in an opposite direction after acute or chronic exposure to Aβ. This demonstrates that species and strains react differently possibly even within the same genus. However, the majority of OTUs could not be classified to these lower taxonomic levels. This highlights the need for optimization of reference databases for taxonomic assignment especially if pre- or probiotic treatments should emerge as an option for ameliorating AD pathology. Namely, such therapies would have to target specific bacterial species or strains which would first have to be identified with high certainty in 16S marker-gene survey studies.

In conclusion, to the best of our knowledge, we provide first evidence that acute *in vivo* administration of Aβ is associated with a shift in gut microbiota composition. Our study does not answer the question if this is the factor that leads to long-term intestinal bacteria alterations in the context of AD. However, we suggest that increased exposure to the Aβ peptide might be an early trigger of a process that ends in a vicious circle of exacerbated AD pathology promoting impaired gut homeostasis and vice versa.

## Data Availability Statement

The raw sequencing data generated in this study have been deposited in the NCBI SRA data base under the accession number PRJNA627235.

## Ethics Statement

The animal study was reviewed and approved by LUA Rhineland-Palatinate, Germany.

## Author Contributions

KE conceived the project and designed the experiments. MS, AM, and KC performed the experiments. HT and CO conducted bioinformatics data analysis. MS and HT wrote the manuscript. KE, TH, SG, MS, and HT edited the manuscript. KE and SG supervised the study. All authors have read and approved the final manuscript.

## Conflict of Interest

HT was employed by Fresenius Kabi Deutschland GmbH at the time the research was conducted. The remining authors declare that the research was conducted in the absence of any commercial or financial relationships that could be construed as a potential conflict of interest.

## Footnotes

1 https://sourceforge.net/projects/bbmap/

## Abbreviations

A β: amyloid- β peptide
AD: Alzheimer’s disease
CFU: colony forming units
Sc: Scrambled
5xFAD: Alzheimer’s disease mouse model
OTU: operational taxonomic unit.
