# Supplementary material for: Impact of Acute and Chronic Amyloid-β Peptide Exposure on Gut Microbial Commensals in the Mouse
Source: Front Microbiol. 2020 May 20;11:1008. doi: 10.3389/fmicb.2020.01008 (PMC7251927; doi:10.3389/fmicb.2020.01008)
Supplement: Supplementary file 1 [file Data_Sheet_1.docx]

Supplementary Material

**Table S1. Alpha diversity measures for the negative control sample**

| **Shannon index** | **Chao index** |
| --- | --- |
| 3.38 | 113.87 |

**
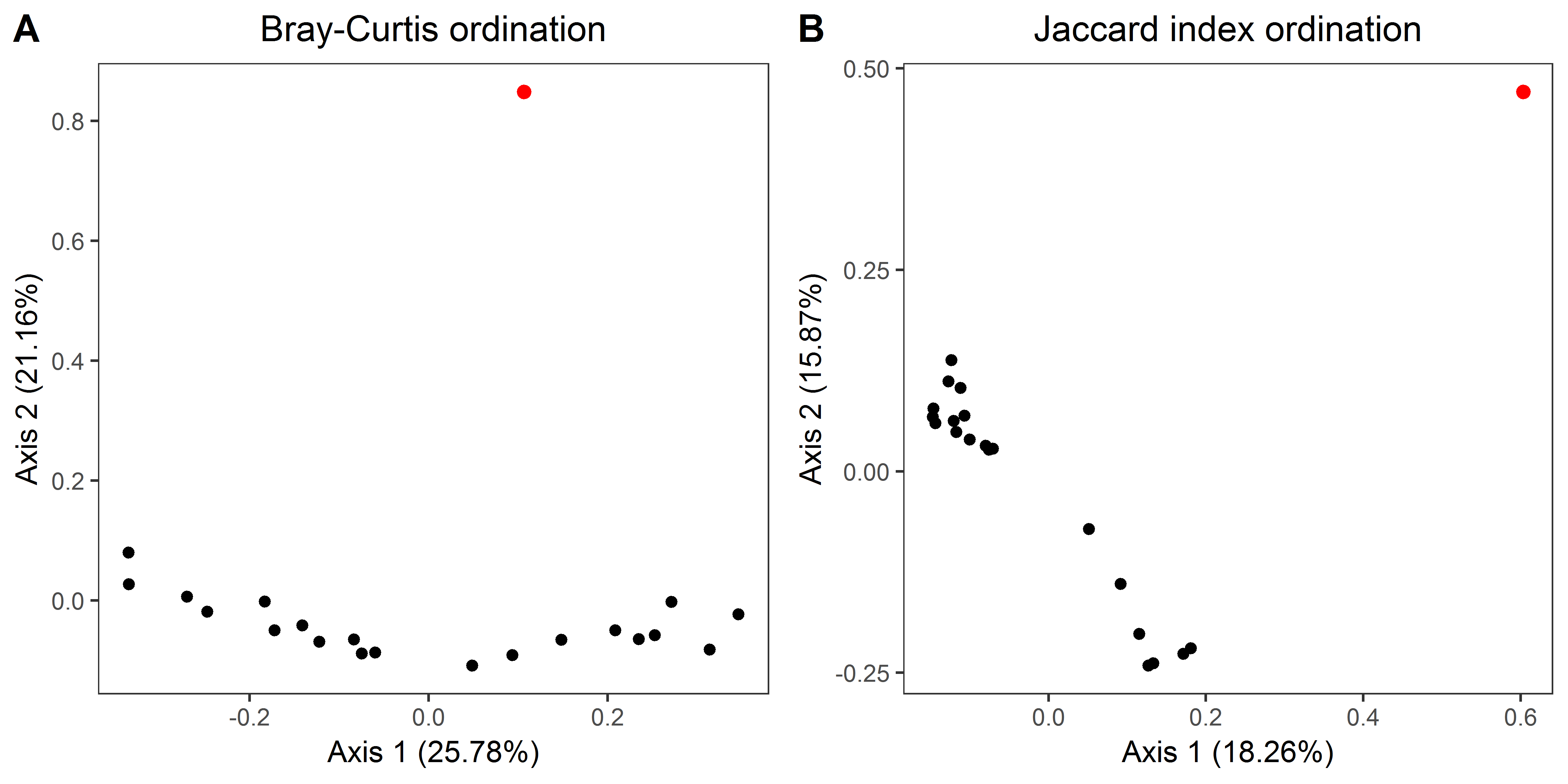
**

**Figure S1. Unconstrained ordination analysis of all experimental groups including negative control.** Ordination analysis was performed based on the **(A)** Bray-Cutis dissimilarity or **(B)** binary Jaccard distance. The negative control sample appears as a red dot on the plots. The amount of dispersion in sample scores (inertia) in percent captured by each multivariate dimension is given in brackets on the respective axis.

**
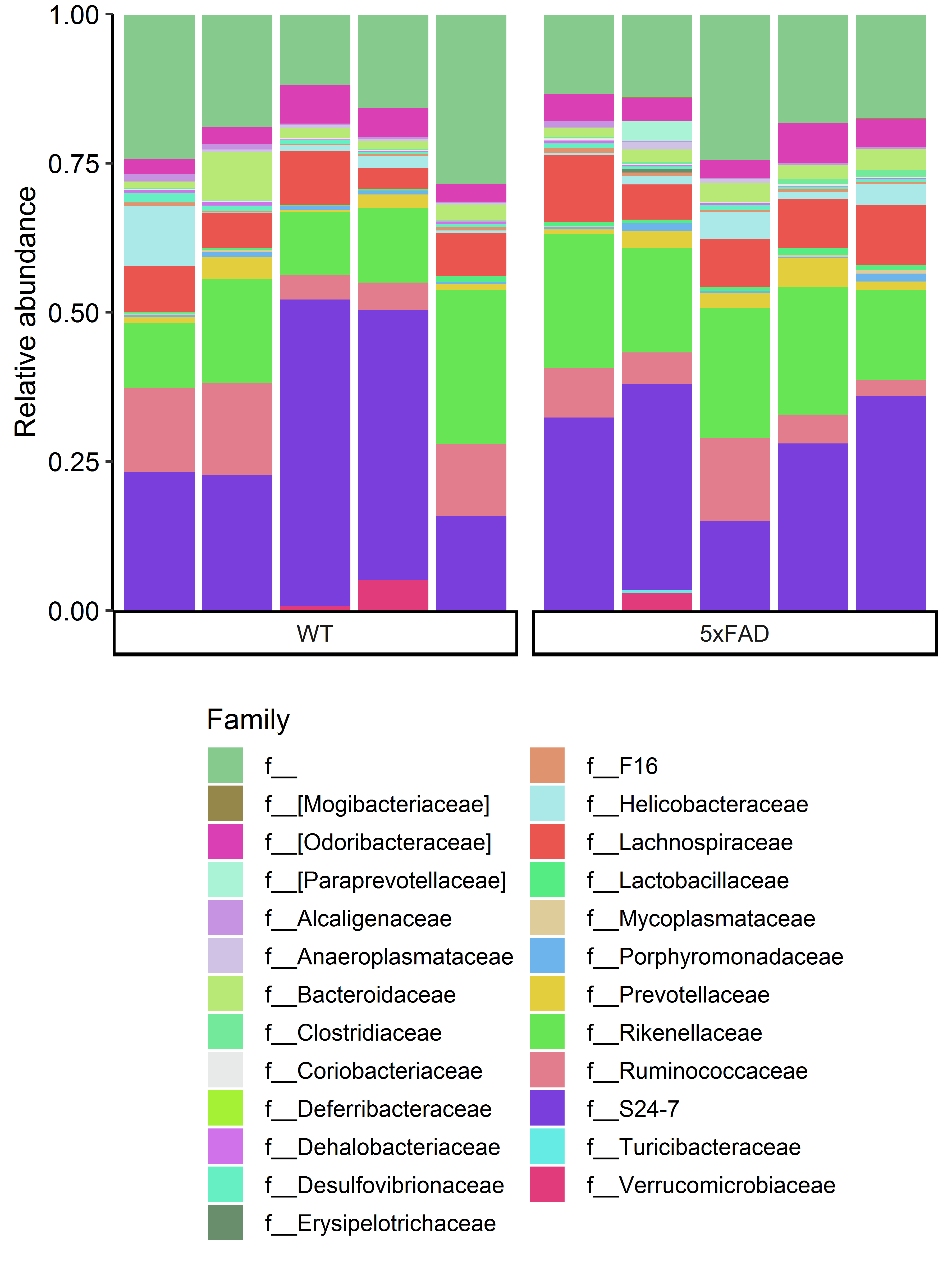
**

**Figure S2. Community composition of wildtype and 5xFAD animals at the family level.** Each bar shows the relative abundance of bacterial families for individual animals.

**
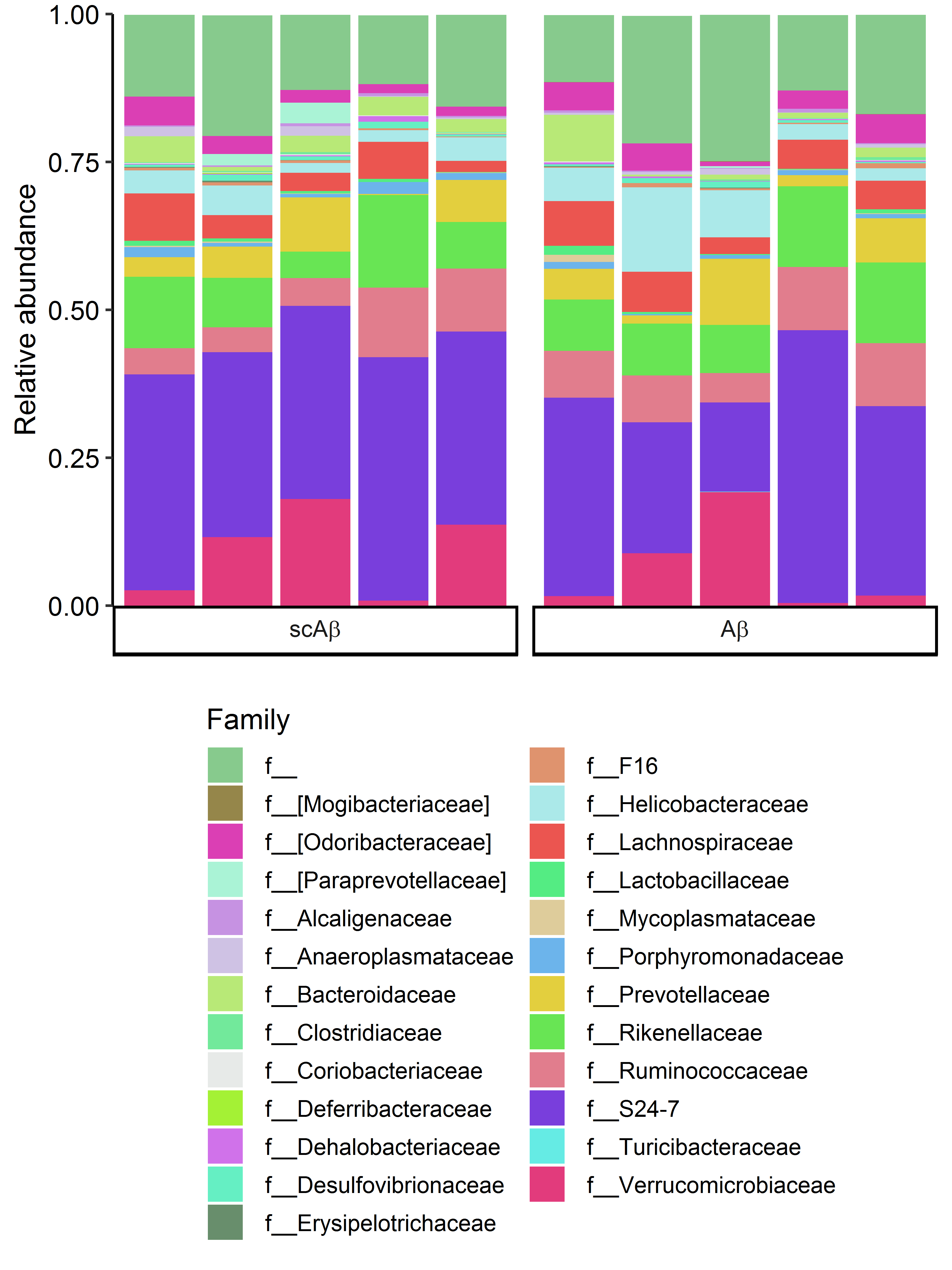
**

**Figure S3. Community composition of scrambled Aβ or Aβ-fed animals at the family level.** Each bar shows the relative abundance of bacterial families for individual animals.

**Table S2 Differentially abundant operational taxonomic units (OTU) in 5xFAD animals relative to wildtype controls.** The taxonomy classification for each OTU, the fold change (FC) together with the corresponding adjusted p-value (Benjamini-Hochberg method) are reported. FC values below 0 indicate OTUs with significantly lower abundance in 5xFAD animals whereas positive FC values correspond to enriched OTUs in the 5xFAD group.

| **Taxonomy** | **Log2 fold change** | **Adj.**  **p-value** |
| --- | --- | --- |
| p__Firmicutes; c__Clostridia; o__Clostridiales; f__Lachnospiraceae | -27.93 | 2.96E-17 |
| p__Firmicutes; c__Clostridia; o__Clostridiales; f__Lachnospiraceae | 25.01 | 5.15E-14 |
| p__Firmicutes; c__Clostridia; o__Clostridiales | 25.09 | 5.15E-14 |
| p__Firmicutes; c__Clostridia; o__Clostridiales | -24.67 | 7.96E-14 |
| p__Firmicutes; c__Clostridia; o__Clostridiales; f__Ruminococcaceae; g__Oscillospira | 24.73 | 7.96E-14 |
| p__Firmicutes; c__Clostridia; o__Clostridiales | 24.54 | 9.32E-14 |
| p__Firmicutes; c__Clostridia; o__Clostridiales | -24.26 | 1.73E-13 |
| p__Firmicutes; c__Clostridia; o__Clostridiales; f__Ruminococcaceae | -24.13 | 2.15E-13 |
| p__Firmicutes; c__Clostridia; o__Clostridiales; f__Ruminococcaceae; g__Ruminococcus | -24 | 2.24E-13 |
| p__Firmicutes; c__Clostridia; o__Clostridiales; f__Lachnospiraceae | 24.04 | 2.24E-13 |
| p__Bacteroidetes; c__Bacteroidia; o__Bacteroidales; f__S24-7 | -24.02 | 2.24E-13 |
| p__Firmicutes; c__Clostridia; o__Clostridiales | -23.91 | 2.38E-13 |
| p__Bacteroidetes; c__Bacteroidia; o__Bacteroidales; f__S24-7 | -23.77 | 3.16E-13 |
| p__Firmicutes; c__Clostridia; o__Clostridiales; f__Lachnospiraceae | -23.74 | 3.23E-13 |
| p__Firmicutes; c__Bacilli; o__Turicibacterales; f__Turicibacteraceae; g__Turicibacter | 23.52 | 5.40E-13 |
| p__Firmicutes; c__Clostridia; o__Clostridiales; f__Clostridiaceae; g__SMB53 | 23.46 | 5.95E-13 |
| p__Firmicutes; c__Clostridia; o__Clostridiales; f__Ruminococcaceae; g__Oscillospira | -23.36 | 6.97E-13 |
| p__Firmicutes; c__Clostridia; o__Clostridiales; o__Clostridiales; o__Clostridiales | -23.36 | 6.97E-13 |
| p__Firmicutes; c__Clostridia; o__Clostridiales; f__Ruminococcaceae; g__Oscillospira | -23.19 | 1.01E-12 |
| p__Bacteroidetes; c__Bacteroidia; o__Bacteroidales; f__S24-7 | -23 | 1.38E-12 |
| p__Firmicutes; c__Clostridia; o__Clostridiales; f__Ruminococcaceae; g__Oscillospira | -23.03 | 1.38E-12 |
| p__Firmicutes; c__Clostridia; o__Clostridiales; f__Ruminococcaceae | -23.01 | 1.38E-12 |
| p__Firmicutes; c__Clostridia; o__Clostridiales; f__Lachnospiraceae | 23.02 | 1.38E-12 |
| p__Firmicutes; c__Erysipelotrichi; o__Erysipelotrichales; f__Erysipelotrichaceae; g__Allobaculum | 22.96 | 1.41E-12 |
| p__Firmicutes; c__Clostridia; o__Clostridiales | -22.98 | 1.41E-12 |
| p__Firmicutes; c__Clostridia; o__Clostridiales | -22.92 | 1.50E-12 |
| p__Firmicutes; c__Erysipelotrichi; o__Erysipelotrichales; f__Erysipelotrichaceae; g__Allobaculum | 22.71 | 2.46E-12 |
| p__Firmicutes; c__Clostridia; o__Clostridiales; f__Lachnospiraceae | 22.59 | 3.15E-12 |
| p__Firmicutes; c__Clostridia; o__Clostridiales | -22.59 | 3.15E-12 |
| p__Firmicutes; c__Clostridia; o__Clostridiales; f__Lachnospiraceae; g__[Ruminococcus]; s__Ruminococcus gnavus | 22.49 | 3.89E-12 |
| p__Bacteroidetes; c__Bacteroidia; o__Bacteroidales; f__Bacteroidaceae; g__Bacteroides; s__Bacteroides acidifaciens | 22.46 | 4.08E-12 |
| p__Firmicutes; c__Bacilli; o__Lactobacillales; f__Lactobacillaceae; g__Lactobacillus; s__Lactobacillus reuteri | 22.41 | 4.50E-12 |
| p__Bacteroidetes; c__Bacteroidia; o__Bacteroidales; f__Rikenellaceae; g__Alistipes; s__Alistipes massiliensis | -22.29 | 5.87E-12 |
| p__Firmicutes; c__Clostridia; o__Clostridiales | -22.26 | 5.88E-12 |
| p__Firmicutes; c__Clostridia; o__Clostridiales | 22.27 | 5.88E-12 |
| p__Proteobacteria; c__Epsilonproteobacteria; o__Campylobacterales; f__Helicobacteraceae; g__Helicobacter; s__Helicobacter hepaticus | -22.26 | 5.88E-12 |
| p__Firmicutes; c__Clostridia; o__Clostridiales; f__Ruminococcaceae; g__Oscillospira | -22.23 | 6.05E-12 |
| p__Firmicutes; c__Erysipelotrichi; o__Erysipelotrichales; f__Erysipelotrichaceae; g__Allobaculum | 22.17 | 6.90E-12 |
| p__Firmicutes; c__Clostridia; o__Clostridiales; f__Lachnospiraceae | 22.14 | 7.22E-12 |
| p__Firmicutes; c__Clostridia; o__Clostridiales | -22.06 | 8.80E-12 |
| p__Bacteroidetes; c__Bacteroidia; o__Bacteroidales; f__Rikenellaceae; g__Alistipes; s__Alistipes massiliensis | 22 | 9.66E-12 |
| p__Firmicutes; c__Clostridia; o__Clostridiales; f__Ruminococcaceae; g__Oscillospira | -21.99 | 9.82E-12 |
| p__Firmicutes; c__Clostridia; o__Clostridiales; f__Lachnospiraceae | -21.97 | 1.03E-11 |
| p__Firmicutes; c__Clostridia; o__Clostridiales | -21.95 | 1.04E-11 |
| p__Firmicutes; c__Clostridia; o__Clostridiales; f__Lachnospiraceae | 21.93 | 1.06E-11 |
| p__Firmicutes; c__Clostridia; o__Clostridiales | -21.88 | 1.18E-11 |
| p__Firmicutes; c__Clostridia; o__Clostridiales | 21.83 | 1.28E-11 |
| p__Firmicutes; c__Clostridia; o__Clostridiales | -21.82 | 1.32E-11 |
| p__Bacteroidetes; c__Bacteroidia; o__Bacteroidales | -21.81 | 1.33E-11 |
| p__Firmicutes; c__Clostridia; o__Clostridiales | -21.75 | 1.50E-11 |
| p__Firmicutes; c__Clostridia; o__Clostridiales | 21.67 | 1.77E-11 |
| p__Firmicutes; c__Clostridia; o__Clostridiales; f__Ruminococcaceae; g__Ruminococcus | -21.65 | 1.84E-11 |
| p__Firmicutes; c__Clostridia; o__Clostridiales | -21.59 | 2.08E-11 |
| p__Firmicutes; c__Clostridia; o__Clostridiales | -21.57 | 2.17E-11 |
| p__Firmicutes; c__Clostridia; o__Clostridiales | 21.55 | 2.18E-11 |
| p__Firmicutes; c__Clostridia; o__Clostridiales; f__Ruminococcaceae; g__Oscillospira | -21.52 | 2.32E-11 |
| p__Firmicutes; c__Clostridia; o__Clostridiales | -21.52 | 2.32E-11 |
| p__Firmicutes; c__Clostridia; o__Clostridiales | -21.42 | 2.71E-11 |
| p__Cyanobacteria; c__Chloroplast; o__Streptophyta | 21.41 | 2.71E-11 |
| p__Firmicutes; c__Clostridia; o__Clostridiales | 21.43 | 2.71E-11 |
| p__Bacteroidetes; c__Bacteroidia; o__Bacteroidales; f__S24-7 | 21.42 | 2.71E-11 |
| p__Firmicutes; c__Clostridia; o__Clostridiales; f__Ruminococcaceae; g__Ruminococcus | 21.42 | 2.71E-11 |
| p__Bacteroidetes; c__Bacteroidia; o__Bacteroidales; f__Rikenellaceae; g__Alistipes; s__Alistipes massiliensis | 21.38 | 2.91E-11 |
| p__Firmicutes; c__Clostridia; o__Clostridiales | 21.37 | 2.92E-11 |
| p__Firmicutes; c__Clostridia; o__Clostridiales | -21.37 | 2.96E-11 |
| p__Firmicutes; c__Clostridia; o__Clostridiales | 21.35 | 3.01E-11 |
| p__Firmicutes; c__Clostridia; o__Clostridiales | -21.35 | 3.02E-11 |
| p__Firmicutes; c__Clostridia; o__Clostridiales; f__Ruminococcaceae; g__Ruminococcus | 21.32 | 3.10E-11 |
| p__Firmicutes; c__Clostridia; o__Clostridiales | 21.28 | 3.33E-11 |
| p__Firmicutes; c__Clostridia; o__Clostridiales | -21.28 | 3.41E-11 |
| p__Firmicutes; c__Clostridia; o__Clostridiales | -21.24 | 3.63E-11 |
| p__Firmicutes; c__Clostridia; o__Clostridiales; f__Lachnospiraceae | 21.23 | 3.63E-11 |
| p__Firmicutes; c__Clostridia; o__Clostridiales | 21.22 | 3.63E-11 |
| p__Firmicutes; c__Clostridia; o__Clostridiales; f__Lachnospiraceae | 21.22 | 3.63E-11 |
| p__Bacteroidetes; c__Bacteroidia; o__Bacteroidales; f__Rikenellaceae; g__Alistipes; s__Alistipes massiliensis | -21.22 | 3.67E-11 |
| p__Firmicutes; c__Clostridia; o__Clostridiales | 21.19 | 3.77E-11 |
| p__Firmicutes; c__Clostridia; o__Clostridiales; f__Ruminococcaceae; g__Ruminococcus | -21.19 | 3.78E-11 |
| p__Bacteroidetes; c__Bacteroidia; o__Bacteroidales; f__Rikenellaceae; g__Alistipes; s__Alistipes massiliensis | 21.17 | 3.91E-11 |
| p__Bacteroidetes; c__Bacteroidia; o__Bacteroidales; f__Rikenellaceae; g__Alistipes; s__Alistipes massiliensis | 21.14 | 4.14E-11 |
| p__Bacteroidetes; c__Bacteroidia; o__Bacteroidales | 21.05 | 4.97E-11 |
| p__Firmicutes; c__Clostridia; o__Clostridiales | -21.06 | 4.97E-11 |
| p__Firmicutes; c__Clostridia; o__Clostridiales | 21.04 | 5.03E-11 |
| p__Firmicutes; c__Clostridia; o__Clostridiales | -21.04 | 5.09E-11 |
| p__Firmicutes; c__Clostridia; o__Clostridiales | 21.02 | 5.17E-11 |
| p__Firmicutes; c__Clostridia; o__Clostridiales | -21.02 | 5.17E-11 |
| p__Bacteroidetes; c__Bacteroidia; o__Bacteroidales; f__Bacteroidaceae; g__Bacteroides | 20.98 | 5.48E-11 |
| p__Firmicutes; c__Clostridia; o__Clostridiales; f__Lachnospiraceae | 20.97 | 5.48E-11 |
| p__Firmicutes; c__Clostridia; o__Clostridiales | -20.97 | 5.49E-11 |
| p__Firmicutes; c__Clostridia; o__Clostridiales; f__Lachnospiraceae | -20.98 | 5.49E-11 |
| p__Firmicutes; c__Clostridia; o__Clostridiales; f__Lachnospiraceae | 20.95 | 5.59E-11 |
| p__Bacteroidetes; c__Bacteroidia; o__Bacteroidales; f__Rikenellaceae; g__Alistipes; s__Alistipes massiliensis | 20.9 | 6.18E-11 |
| p__Firmicutes; c__Clostridia; o__Clostridiales | 20.79 | 8.12E-11 |
| p__Bacteroidetes; c__Bacteroidia; o__Bacteroidales; f__Rikenellaceae; g__Alistipes; s__Alistipes massiliensis | 20.76 | 8.54E-11 |
| p__Bacteroidetes; c__Bacteroidia; o__Bacteroidales | 20.66 | 1.07E-10 |
| p__Firmicutes; c__Clostridia; o__Clostridiales | 20.58 | 1.27E-10 |
| p__Firmicutes; c__Clostridia; o__Clostridiales | 20.52 | 1.36E-10 |
| p__Firmicutes; c__Clostridia; o__Clostridiales | 20.5 | 1.50E-10 |
| p__Firmicutes; c__Clostridia; o__Clostridiales; f__Ruminococcaceae; g__Ruminococcus | 20.45 | 1.63E-10 |
| p__Firmicutes; c__Clostridia; o__Clostridiales | 20.45 | 1.63E-10 |
| p__Firmicutes; c__Clostridia; o__Clostridiales | 20.43 | 1.70E-10 |
| p__Firmicutes; c__Clostridia; o__Clostridiales | 19.82 | 6.26E-10 |
| p__Bacteroidetes; c__Bacteroidia; o__Bacteroidales; f__S24-7 | -9.49 | 9.01E-05 |
| p__Firmicutes; c__Clostridia; o__Clostridiales | 10.94 | 0.003137 |
| p__Firmicutes; c__Clostridia; o__Clostridiales; f__Ruminococcaceae; g__Oscillospira; | 10.77 | 0.003848 |
| p__Proteobacteria; c__Alphaproteobacteria; o__RF32;;; | -10.01 | 0.00635 |
| p__Firmicutes; c__Clostridia; o__Clostridiales;;; | 10.31 | 0.00664 |
| p__Tenericutes; c__Mollicutes; o__Anaeroplasmatales; f__Anaeroplasmataceae; g__Anaeroplasma | 9.99 | 0.007711 |
| p__Bacteroidetes; c__Bacteroidia; o__Bacteroidales; f__[Paraprevotellaceae]; g__[Prevotella] | 7.91 | 0.010456 |
| p__Firmicutes; c__Clostridia; o__Clostridiales; f__Clostridiaceae; g__Candidatus Arthromitus | 2.91 | 0.03776 |

**Table S3. Differentially abundant operational taxonomic units (OTU) in amyloid-β (Aβ) relative to scrambled amyloid beta (scAβ)-fed animals.** The taxonomy classification for each OTU, the fold change (FC) together with the corresponding adjusted p-value (Benjamini-Hochberg method) are reported. FC below 0 indicate OTUs with significantly lower abundance in Aβ animals whereas positive FC values correspond to enriched OTUs in the Aβ group.

| **Taxonomy** | **Log2 fold change** | **Adj. p-value** |
| --- | --- | --- |
| p__Firmicutes; c__Clostridia; o__Clostridiales; f__Lachnospiraceae; | 24.24 | 1.72E-16 |
| p__Tenericutes; c__Mollicutes; o__Anaeroplasmatales; f__Anaeroplasmataceae; g__Anaeroplasma | -26.96 | 1.84E-16 |
| p__Firmicutes; c__Clostridia; o__Clostridiales; f__Ruminococcaceae; g__Oscillospira | -24.99 | 3.50E-14 |
| p__Firmicutes; c__Clostridia; o__Clostridiales | 24.8 | 4.45E-14 |
| p__Firmicutes; c__Clostridia; o__Clostridiales; f__Ruminococcaceae; g__Ruminococcus | 24.68 | 4.98E-14 |
| p__Firmicutes; c__Clostridia; o__Clostridiales; f__Lachnospiraceae; g__[Ruminococcus]; s__Ruminococcus gnavus | -24.11 | 5.89E-14 |
| p__Firmicutes; c__Clostridia; o__Clostridiales | 23.75 | 7.10E-14 |
| p__Firmicutes; c__Clostridia; o__Clostridiales; f__Ruminococcaceae; g__Oscillospira | -23.78 | 7.10E-14 |
| p__Firmicutes; c__Clostridia; o__Clostridiales; f__Lachnospiraceae | 23.94 | 7.10E-14 |
| p__Firmicutes; c__Clostridia; o__Clostridiales; f__Lachnospiraceae | -22.93 | 2.22E-13 |
| p__Firmicutes; c__Clostridia; o__Clostridiales; f__Ruminococcaceae; g__Oscillospira | 22.81 | 2.89E-13 |
| p__Firmicutes; c__Clostridia; o__Clostridiales | -23.38 | 6.71E-13 |
| p__Firmicutes; c__Clostridia; o__Clostridiales | 22.02 | 1.22E-12 |
| p__Bacteroidetes; c__Bacteroidia; o__Bacteroidales; f__S24-7 | -22.79 | 2.60E-12 |
| p__Bacteroidetes; c__Bacteroidia; o__Bacteroidales; f__Rikenellaceae | -22.71 | 2.99E-12 |
| p__Firmicutes; c__Clostridia; o__Clostridiales; f__Lachnospiraceae | -22.58 | 3.83E-12 |
| p__Firmicutes; c__Clostridia; o__Clostridiales; f__Ruminococcaceae; g__Ruminococcus | -22.55 | 3.94E-12 |
| p__Firmicutes; c__Clostridia; o__Clostridiales; f__Lachnospiraceae | -22.48 | 4.45E-12 |
| p__Firmicutes; c__Clostridia; o__Clostridiales | -22.38 | 5.50E-12 |
| p__Firmicutes; c__Clostridia; o__Clostridiales | -22.09 | 1.07E-11 |
| p__Firmicutes; c__Clostridia; o__Clostridiales | -22.06 | 1.10E-11 |
| p__Firmicutes; c__Clostridia; o__Clostridiales; f__Ruminococcaceae; g__Oscillospira | -22.01 | 1.17E-11 |
| p__Firmicutes; c__Clostridia; o__Clostridiales; f__Ruminococcaceae | -21.89 | 1.51E-11 |
| p__Firmicutes; c__Clostridia; o__Clostridiales | -21.87 | 1.52E-11 |
| p__Firmicutes; c__Clostridia; o__Clostridiales | -21.63 | 2.42E-11 |
| p__Firmicutes; c__Clostridia; o__Clostridiales | 21.64 | 2.42E-11 |
| p__Firmicutes; c__Clostridia; o__Clostridiales | 21.59 | 2.56E-11 |
| p__Firmicutes; c__Clostridia; o__Clostridiales | -21.53 | 2.93E-11 |
| p__Firmicutes; c__Clostridia; o__Clostridiales | 21.46 | 3.27E-11 |
| p__Firmicutes; c__Clostridia; o__Clostridiales; f__Lachnospiraceae | -21.36 | 4.14E-11 |
| p__Firmicutes; c__Clostridia; o__Clostridiales; f__Ruminococcaceae | -21.34 | 4.16E-11 |
| p__Firmicutes; c__Clostridia; o__Clostridiales; f__Ruminococcaceae | -21.32 | 4.27E-11 |
| p__Firmicutes; c__Clostridia; o__Clostridiales; f__Ruminococcaceae; g__Ruminococcus | 21.23 | 4.90E-11 |
| p__Firmicutes; c__Clostridia; o__Clostridiales; f__Ruminococcaceae; g__Oscillospira | -21.24 | 4.90E-11 |
| p__Firmicutes; c__Clostridia; o__Clostridiales; f__Ruminococcaceae | -21.2 | 5.19E-11 |
| p__Firmicutes; c__Clostridia; o__Clostridiales; f__Lachnospiraceae | -21.1 | 6.11E-11 |
| p__Firmicutes; c__Clostridia; o__Clostridiales; | -21.12 | 6.11E-11 |
| p__Firmicutes; c__Clostridia; o__Clostridiales; f__Lachnospiraceae | -21.08 | 6.42E-11 |
| p__Firmicutes; c__Clostridia; o__Clostridiales; f__Lachnospiraceae; | 20.97 | 7.70E-11 |
| p__Bacteroidetes; c__Bacteroidia; o__Bacteroidales; f__S24-7 | -20.97 | 7.88E-11 |
| p__Firmicutes; c__Clostridia; o__Clostridiales; f__Ruminococcaceae; g__Oscillospira | 20.95 | 7.92E-11 |
| p__Firmicutes; c__Clostridia; o__Clostridiales; f__Lachnospiraceae; g__[Ruminococcus]; s__Ruminococcus gnavus | -20.94 | 7.96E-11 |
| p__Bacteroidetes; c__Bacteroidia; o__Bacteroidales | -20.89 | 8.55E-11 |
| p__Firmicutes; c__Clostridia; o__Clostridiales | 20.89 | 8.55E-11 |
| p__Firmicutes; c__Clostridia; o__Clostridiales | 20.85 | 9.09E-11 |
| p__Firmicutes; c__Clostridia; o__Clostridiales | -20.77 | 1.07E-10 |
| p__Bacteroidetes; c__Bacteroidia; o__Bacteroidales; f__S24-7 | 20.71 | 1.21E-10 |
| p__Firmicutes; c__Clostridia; o__Clostridiales | 20.66 | 1.33E-10 |
| p__Firmicutes; c__Clostridia; o__Clostridiales | 20.58 | 1.49E-10 |
| p__Cyanobacteria; c__Chloroplast; o__Streptophyta | 20.55 | 1.67E-10 |
| p__Firmicutes; c__Clostridia; o__Clostridiales; f__Ruminococcaceae; g__Ruminococcus | 9.5 | 0.003558 |
| p__Firmicutes; c__Clostridia; o__Clostridiales; f__Ruminococcaceae; g__Oscillospira | 8.73 | 0.009744 |
| p__Firmicutes; c__Clostridia; o__Clostridiales | -8.32 | 0.01723 |
| p__Firmicutes; c__Bacilli; o__Lactobacillales; f__Lactobacillaceae; g__Lactobacillus;  s__Lactobacillus reuteri | -7.92 | 0.02619 |
| p__Firmicutes; c__Clostridia; o__Clostridiales; f__Lachnospiraceae | -8.06 | 0.02619 |


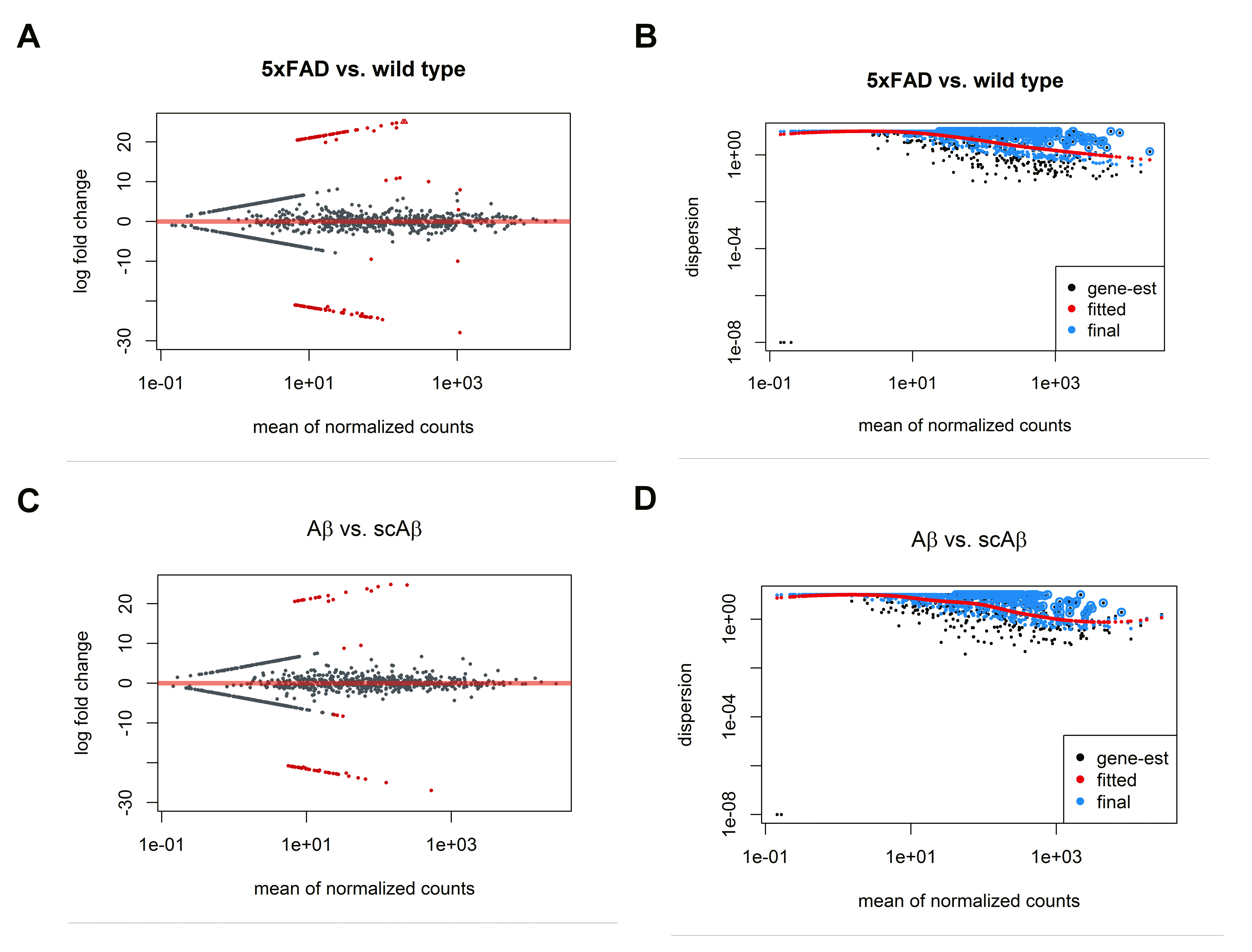


**Figure S4. Diagnostic plots for the differential abundance analysis performed with DESeq2.** The relationship between the mean normalized bacterial abundance and log2 fold change is shown in panel A for 5xFAD mice relative to wild type mice and panel C for Aβ-fed wild type mice relative to animals receiving scrambled Aβ (scAβ). Fold changes with an adjusted p-value less than 0.05 are colored red. The relationship between the dispersion and mean parameters of the negative binomial distributions used to estimate log2 fold changes is shown in panels B and D. Dispersions estimates directly obtained from the data appear as black dots, the red line corresponds to the fitted values using local regression. The blue dots correspond to the final estimates of dispersion after shrinkage.
